# Supplementary figures and images for: SNARE mimicry by the CD225 domain of IFITM3 enables regulation of homotypic late endosome fusion
Source: bioRxiv. 2024 Nov 22:2024.08.07.607021. Preprint. [Version 2] doi: 10.1101/2024.08.07.607021 (PMC12478428; doi:10.1101/2024.08.07.607021)

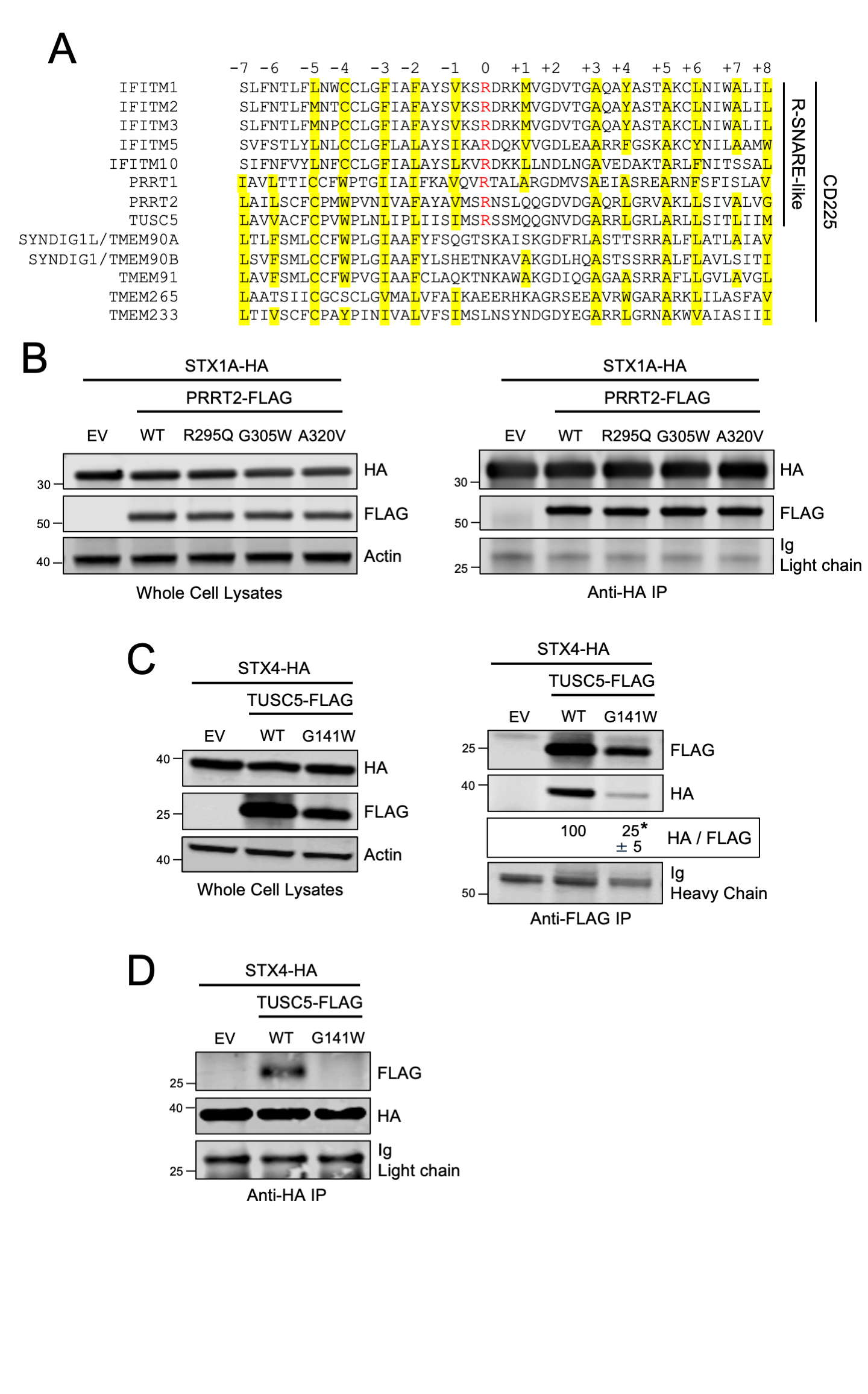

Supplement: Supplement 2 — Figure EV1: An R-SNARE-like motif is semi-conserved among human CD225 family members. (A) Human CD225 proteins were aligned and trimmed to a 53-residue stretch of sequence covering the SNARE motif. Proteins are grouped by whether they contain an arginine at the central “0” layer (R-SNARE-like) or not. Surrounding heptad repeats of hydrophobic residues are labeled from “−7” to “+8” in relation to the “0” layer, and sites highlighted in yellow correspond to hydrophobic residues. (B) Left: HEK293T cells were co-transfected with STX1A-HA and either PRRT2-FLAG (WT, R295Q, G305W, or A320V) or Empty Vector. SDS-PAGE and immunoblotting were performed with anti-HA and anti-FLAG in whole cell lysates. Anti-actin was used as loading control. Right: From co-transfected cells, STX1A-HA was immunoprecipitated with anti-HA followed by SDS-PAGE and immunoblotting with anti-HA and anti-FLAG. Immunoglobulin chain was used as loading control. (C) Left: HEK293T cells were co-transfected with STX4-HA and either TUSC5-FLAG (WT or G141W) or Empty Vector. SDS-PAGE and immunoblotting were performed with anti-HA and anti-FLAG in whole cell lysates. Anti-actin was used as loading control. Right: From co-transfected cells, TUSC5-FLAG was immunoprecipitated with anti-FLAG followed by SDS-PAGE and immunoblotting with anti-HA and anti-FLAG. Light chain immunoglobulin chain was used as loading control. The HA/FLAG ratio was calculated for the indicated lanes and shown as mean and standard error (normalized relative to WT, which was set to 100%). Differences that were statistically significant from WT as determined by student’s T test are indicated by (*). Exact p value: p < 0.0001. (D) From co-transfected cells, STX4-HA was immunoprecipitated with anti-HA followed by SDS-PAGE and immunoblotting with anti-FLAG and anti-HA. Light chain immunoglobulin was used as loading control. Numbers and tick marks left of blots indicate position and size (in kilodaltons) of protein standard in ladder. Immunoblot [file media-2.tif]

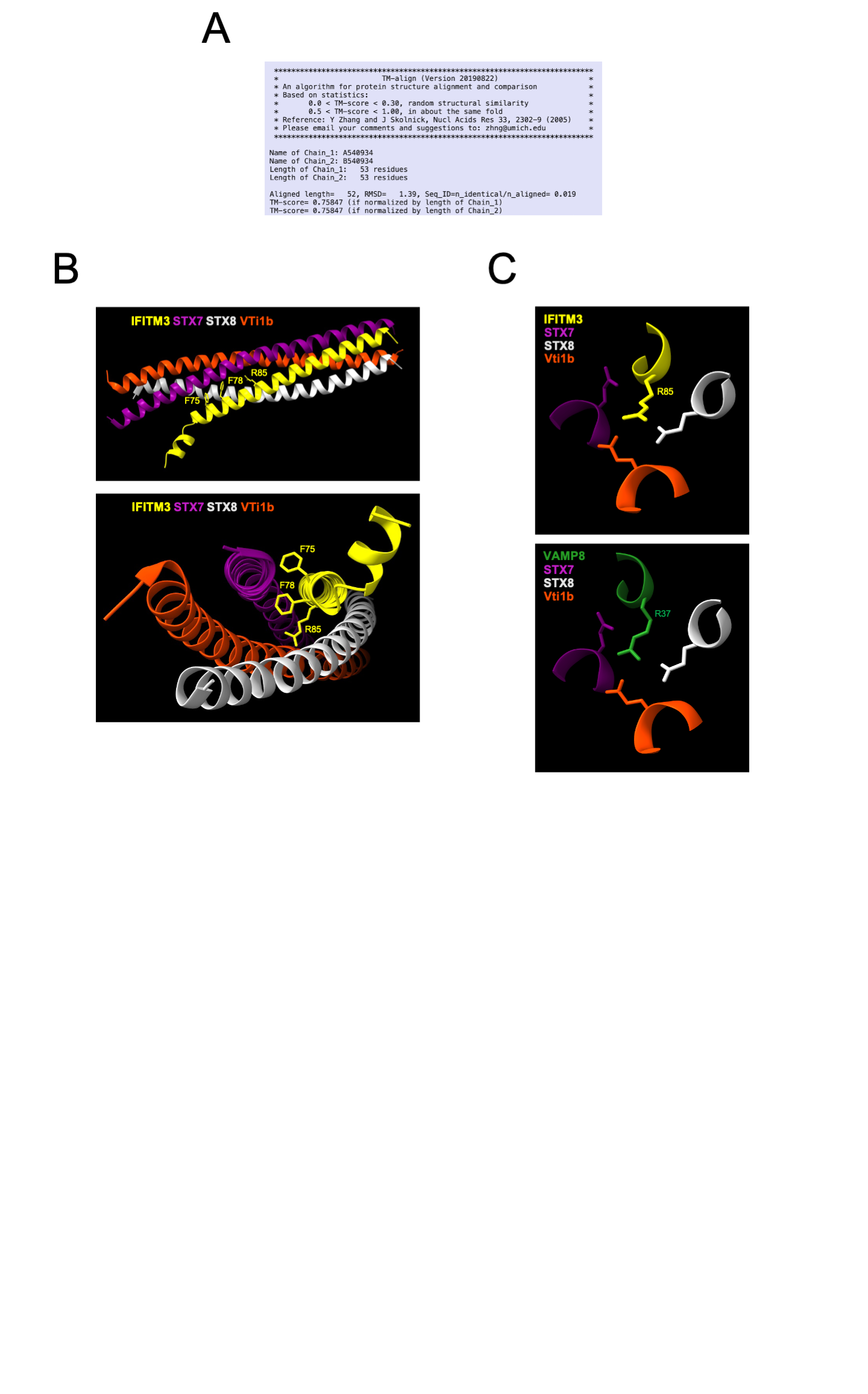

Supplement: Supplement 3 — Figure EV2: IFITM3 contains a motif resembling the alpha helical R-SNARE motif of VAMP8 (A) The R-SNARE-like motif of IFITM3 and the R-SNARE motif of VAMP8 (53 residues each) were compared by predictive protein structure alignment and comparison algorithm TM-align. A TM score of 0.75847 was recorded, indicating that the two regions are likely to adopt the same alpha helical protein fold. (B) Structural prediction of the R-SNARE-like motif of IFITM3 was performed with Alphafold and FATCAT. Residues 55–113 of IFITM3 were modeled against a template of the R-SNARE motif of VAMP8 which was previously crystallized as part of the STX7-STX8-Vti1b-VAMP8 trans-SNARE complex (PDB: 1GL2). VAMP8 was then swapped with the predicted structure of IFITM3 and shown in relation to the coiled-coiled structure formed with STX7, STX8, and Vti1b. Top: the side chains of residues F75, F78, and R85 of IFITM3 are depicted in yellow and labeled. Bottom: alternative view of the side chains of F75, F78, and R85 of IFITM3. (C) Top: examination of the central polar “0” layer of the Q-SNAREs STX7, STX8, Vti1b in relation to the R85 residue in the predicted configuration of IFITM3. Bottom: examination of the central polar “0” layer of the STX7-STX8-Vti1b-VAMP8 complex (PDB: IGL2). [file media-3.tif]

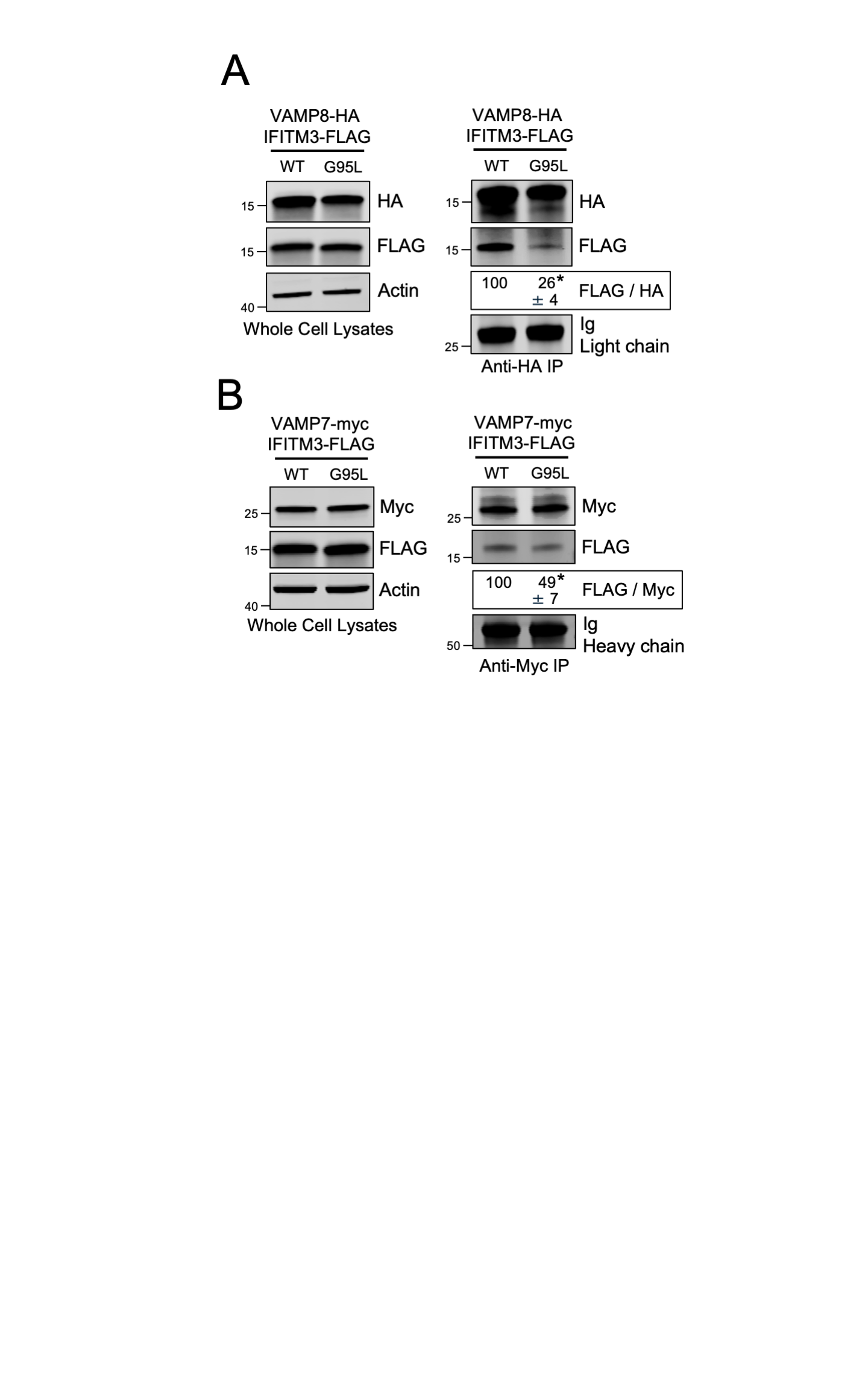

Supplement: Supplement 4 — Figure EV3: IFITM3 interacts with the R-SNAREs VAMP8 and VAMP7 (A) Left: HEK293T cells were co-transfected with VAMP8-HA and IFITM3-FLAG (WT or G95L). SDS-PAGE and immunoblotting were performed with anti-HA and anti-FLAG in whole cell lysates. Anti-actin was used as loading control. Right: From co-transfected cells, VAMP8-HA was immunoprecipitated with anti-HA followed by SDS-PAGE and immunoblotting with anti-HA and anti-FLAG. Light chain immunoglobulin chain was used as loading control. The FLAG/HA ratio was calculated for the indicated lanes and shown as mean and standard error (normalized relative to WT, which was set to 100%). Differences that were statistically significant from WT as determined by student’s T test are indicated by (*). Exact p value: p < 0.0001. (B) Left: HEK293T cells were co-transfected with VAMP7-myc and IFITM3-FLAG (WT or G95L). SDS-PAGE and immunoblotting were performed with anti-myc and anti-FLAG in whole cell lysates. Anti-actin was used as loading control. Right: From co-transfected cells, VAMP7-myc was immunoprecipitated with anti-myc followed by SDS-PAGE and immunoblotting with anti-myc and anti-FLAG. Light chain immunoglobulin chain was used as loading control. The FLAG/Myc ratio was calculated for the indicated lanes and shown as mean and standard error (normalized relative to WT, which was set to 100%). Differences that were statistically significant from WT as determined by student’s T test are indicated by (*). Exact p value: p = 0.0023. Numbers and tick marks left of blots indicate position and size (in kilodaltons) of protein standard in ladder. Immunoblots were performed independently three times, and a representative example is shown. Ig; immunoglobulin. IP; immunoprecipitation. WT; wild-type. [file media-4.tif]

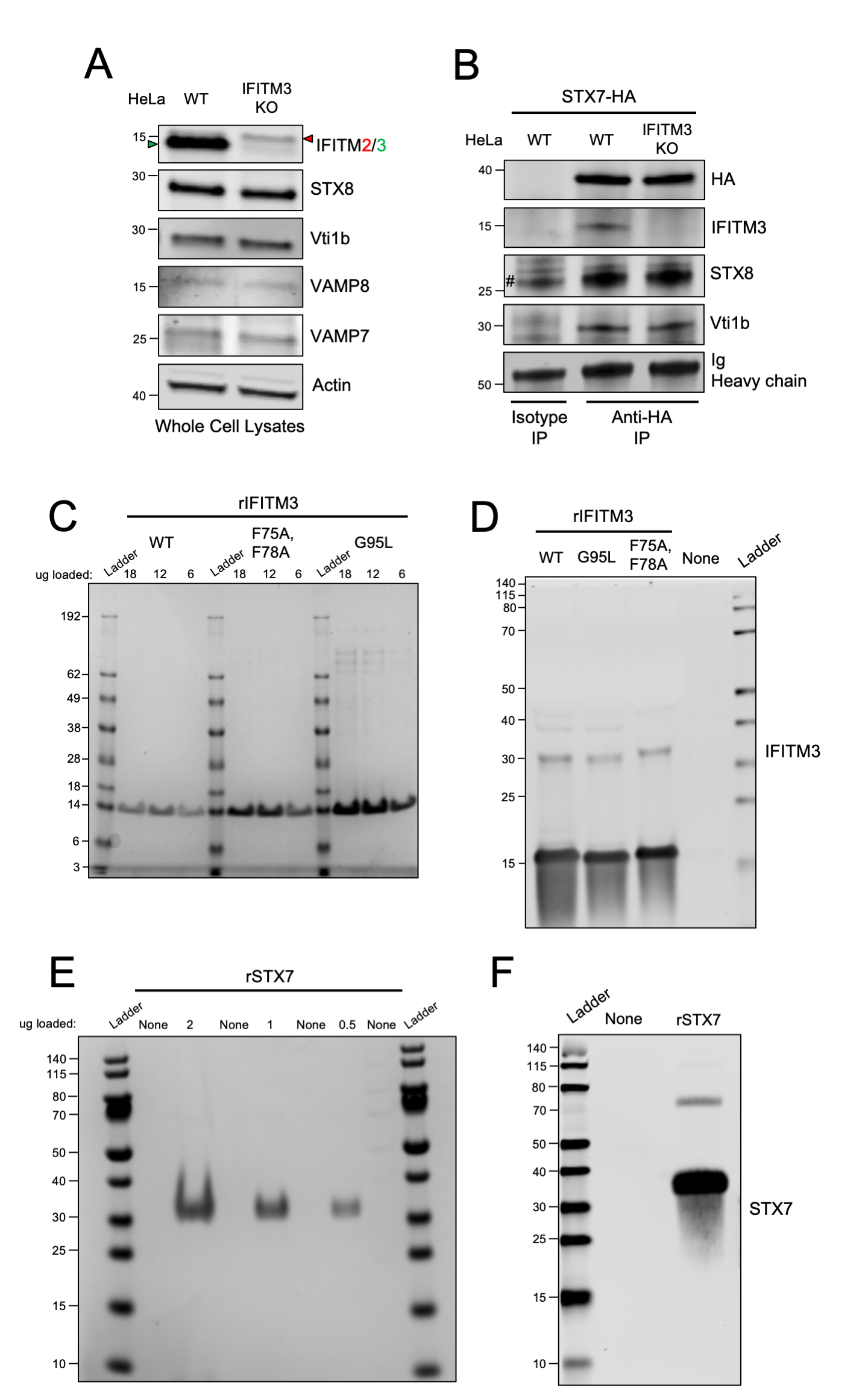

Supplement: Supplement 5 — Figure EV4: IFITM3 does not impair the STX7/STX8 or STX7/Vti1b interactions (A) HeLa cells (WT or IFITM3 KO) were subjected to whole cell lysis, SDS-PAGE, and immunoblotting with anti-IFITM2/3, anti-STX8, anti-Vti1b, anti-VAMP8, anti-VAMP7, and anti-actin. Red and green arrows indicate IFITM3 and IFITM2, respectively, recognized by the anti-IFITM2/3 antibody. (B) STX7-HA was transfected into HeLa (WT or IFITM3 KO) and immunoprecipitated with anti-HA followed by SDS-PAGE and immunoblotting with anti-HA, anti-IFITM3, anti-STX8, and anti-Vti1b. Heavy chain immunoglobulin was used as loading control. An isotype matched antibody was used as a control for immunoprecipitation. The (#) symbol denotes the presence of light chain immunoglobulin. (C) Purified recombinant IFITM3 protein (WT, F75/78A, or G95L) of varying inputs (6, 12 or 18 μg) were subjected to SDS-PAGE and visualized by Coomassie stain. (D) 20 ng of purified recombinant IFITM3 protein (WT, F75/78A, or G95L) were subjected to SDS-PAGE and immunoblotting with anti-IFITM3. (E) Recombinant STX7 of varying inputs (0.5, 1, or 2 μg) were subjected to SDS-PAGE and visualized by Coomassie stain. (F) Recombinant STX7 was subjected to SDS-PAGE and immunoblotting with anti-STX7. Numbers and tick marks left of blots indicate position and size (in kilodaltons) of protein standard in ladder. Immunoblots were performed independently two times and a representative example is shown (for (A) and (B)) or once (for (C), (D), (E), and (F)). Ig; immunoglobulin. IP; immunoprecipitation. EV; Empty Vector. WT; wild-type. r; recombinant. [file media-5.tif]

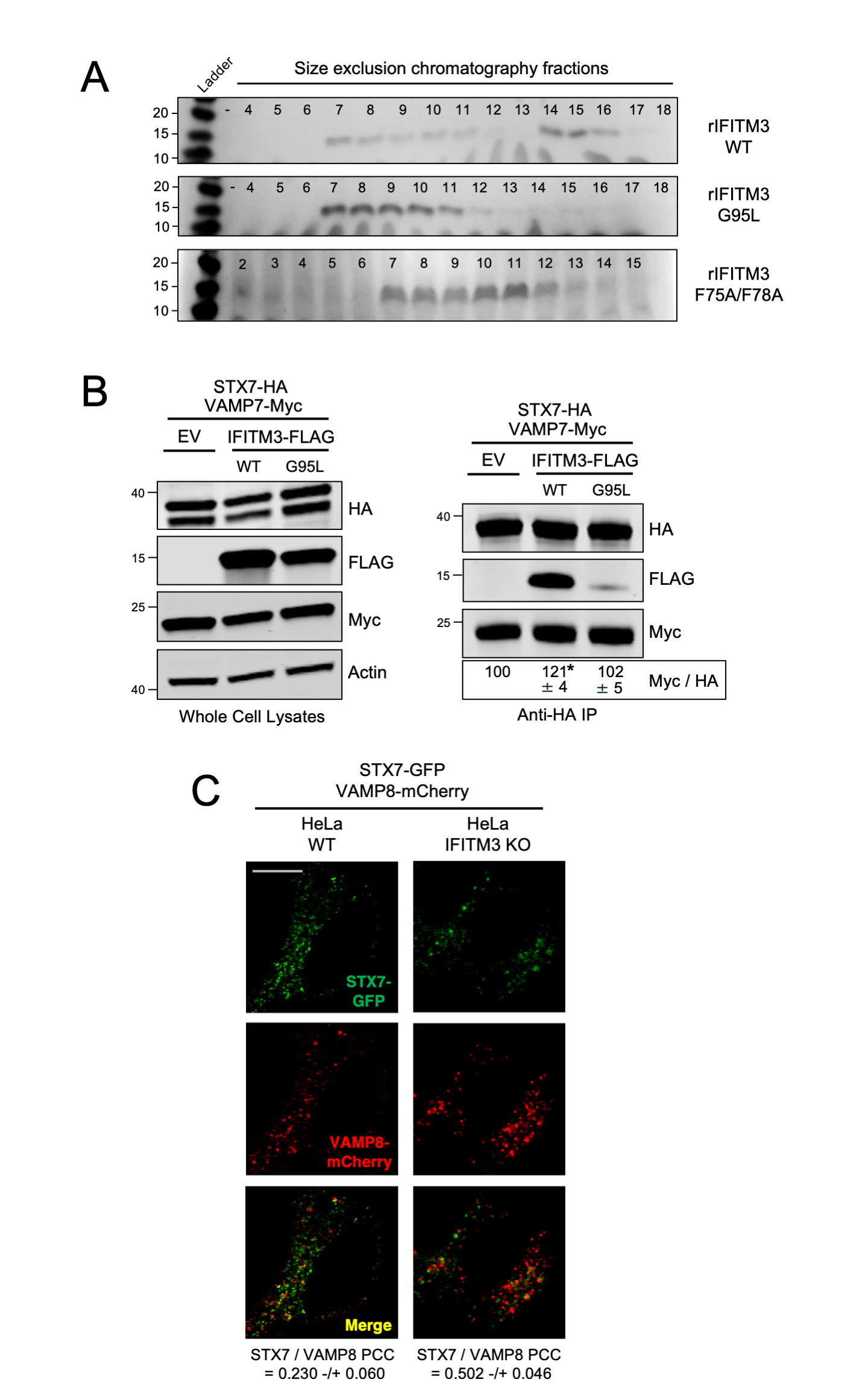

Supplement: Supplement 6 — Figure EV5: IFITM3 does not impair the STX7/VAMP7 interaction (A) Size exclusion chromatography was performed on recombinant IFITM3 proteins (WT, F75/78A, and G95L) and eluted fractions were analyzed by SDS-PAGE and Coomassie staining. 150 μg recombinant protein was loaded as input and fraction numbers correspond to mL volumes eluted from column. (B) Left: HEK293T cells stably expressing Empty Vector, IFITM3 WT-FLAG, or IFITM3 G95L-FLAG were co-transfected with STX7-HA and VAMP7-Myc. Whole cell lysates were subjected to SDS-PAGE and immunoblotting with anti-HA, anti-FLAG, and anti-Myc. Actin was used as a loading control. Right: STX7-HA was immunoprecipitated with anti-HA followed by SDS-PAGE and immunoblotting with anti-HA, anti-FLAG, and anti-Myc. The Myc/HA ratio was calculated for the indicated lanes and shown as mean and standard error (normalized relative to Empty Vector, which was set to 100%). Differences that were statistically significant from Empty Vector as determined by one-way ANOVA are indicated by (*). Exact p values are as follows (from left to right): p = 0.0099, p = 0.8782. Numbers and tick marks left of blots indicate position and size (in kilodaltons) of protein standard in ladder. Immunoblots were performed independently three times, and a representative example is shown (except (A), which was performed once). (C) HeLa (WT or IFITM3 KO) were transfected with STX7-GFP and VAMP8-mCherry and confocal immunofluorescence microscopy was performed. Colocalization between STX7-GFP and VAMP8-mCherry was measured by calculating the Pearson’s Correlation Coefficient using Fiji software. Coefficients were calculated from medial Z-slices from three fields of view containing 5–15 cells per condition and presented as means and standard error. Scale bar = 15 microns. IP; immunoprecipitation. WT; wild-type. r; recombinant. [file media-6.tif]
